# Shoc2/Sur8 Protein Regulates Neurite Outgrowth

**DOI:** 10.1371/journal.pone.0114837

**Published:** 2014-12-16

**Authors:** Gonzalo Leon, Lucia Sanchez-Ruiloba, Andrea Perez-Rodriguez, Teresa Gragera, Natalia Martinez, Silvia Hernandez, Berta Anta, Olga Calero, Carlota A. Garcia-Dominguez, Lara M. Dura, Daniel Peña-Jimenez, Judit Castro, Natasha Zarich, Pilar Sanchez-Gomez, Miguel Calero, Teresa Iglesias, Jose L. Oliva, Jose M. Rojas

**Affiliations:** 1 Unidad de Biología Celular, Unidad Funcional de Investigación de Enfermedades Crónicas, Instituto de Salud Carlos III, 28220 Majadahonda, Madrid, Spain; 2 Instituto de Investigaciones Biomédicas “Alberto Sols,” Consejo Superior de Investigaciones Científicas-Universidad Autónoma de Madrid, 28029 Madrid, Spain; 3 Centro de Investigación Biomédica en Red sobre Enfermedades Neurodegenerativas, CIBERNED, Madrid, Spain; 4 Unidad de Encefalopatías Espongiformes, Unidad Funcional de Investigación de Enfermedades Crónicas, Instituto de Salud Carlos III, 28220 Majadahonda, Madrid, Spain; 5 Unidad de Neuro-Oncología, Unidad Funcional de Investigación de Enfermedades Crónicas, Instituto de Salud Carlos III, 28220 Majadahonda, Madrid, Spain; CSIC/Universidad Autonoma Madrid, Spain

## Abstract

The Shoc2 protein has been implicated in the positive regulation of the Ras-ERK pathway by increasing the functional binding interaction between Ras and Raf, leading to increased ERK activity. Here we found that Shoc2 overexpression induced sustained ERK phosphorylation, notably in the case of EGF stimulation, and Shoc2 knockdown inhibited ERK activation. We demonstrate that ectopic overexpression of human Shoc2 in PC12 cells significantly promotes neurite extension in the presence of EGF, a stimulus that induces proliferation rather than differentiation in these cells. Finally, Shoc2 depletion reduces both NGF-induced neurite outgrowth and ERK activation in PC12 cells. Our data indicate that Shoc2 is essential to modulate the Ras-ERK signaling outcome in cell differentiation processes involved in neurite outgrowth.

## Introduction

PC12 cells, which are derived from a rat pheochromocytoma, have been used classically as a model to address the biological outcomes due to functional differences of the extracellular signal-regulated kinase (ERK) signaling pathway [1], [2]. This cascade, which involves the Raf, MEK and ERK proteins, can be activated in PC12 cells by two growth factors, epidermal growth factor (EGF) [2], and nerve growth factor (NGF) [3], [4], leading to PC12 proliferation and differentiation, respectively. The duration of ERK signaling is responsible for the distinct biological effects of EGF and NGF stimulation on PC12 cells [1], [2]. EGF elicits transient Ras- (and also Rap-) dependent ERK activation [2], yielding PC12 cell proliferation, whereas NGF promotes sustained ERK phosphorylation, which leads to cell differentiation (neurite outgrowth) [2], [5], [6].

Ras proteins (H-, N-, and K-Ras) operate as molecular switches in signal transduction cascades, controlling cell proliferation, differentiation and apoptosis. Ras proteins exist in equilibrium between an active (Ras-GTP) and an inactive (Ras-GDP) state. The action of growth factors increases cellular Ras-GTP levels, enabling interaction of this GTPase with its target protein effectors. Activated Ras stimulates various downstream signaling pathways. A wide range of proteins interact specifically with the Ras-GTP complex, including Raf proteins, members of the Ral-GDS family, PI3K, p120GAP, NF1, MEKK1, Rin1, AF-6, PKC-ξ, and Nore1 [7]. For example, Ras-GTP binds directly to Raf proteins, inducing their translocation to membranes where they are activated, in turn triggering induction of the MEK-ERK cascade. Different scaffold proteins bind several components of the Ras/Raf/MEK/ERK cascade, providing molecular signaling platforms, and can thus regulate the Ras-ERK pathway [8], [9].

One of these scaffold proteins is the suppressor of clear homolog (Shoc2) [10], also known as a suppressor of Ras-8 (Sur8) [11]. Shoc2/Sur8 is a protein conserved in all metazoans; it has numerous leucine-rich repeats and selectively binds members of the Ras family [12]. Fluorescence resonance energy transfer (FRET) imaging and computational modeling studies showed that Shoc2 is essential for Ras-GTP signaling to ERK, because it accelerates the Ras-Raf interaction probably by stabilizing a ternary complex formed by all these proteins [12], [13], [14]. Furthermore, single mutation of Shoc2 causes Noonan-like syndrome, a type of neuro-cardio-facial-cutaneous disorders, by enhancing ERK activation [15]. Other group nonetheless proposes an alternative mechanism of action for Shoc2, by which this scaffold protein forms a ternary complex with the catalytic subunit of protein phosphatase 1 (PP1c) and M-Ras (a Ras family member), which promotes Raf activity by dephosphorylating its S259 inhibitory residue [16], and also forms a complex with SCRIB playing a key role in polarized migration [17].

Based on Shoc2 function as a positive modulator of the ERK-pathway, we hypothesized that this scaffold protein might have a pivotal role in PC12 cell differentiation, a process dependent on sustained ERK activation. Here, we analyzed how ectopic overexpression of Shoc2 affects EGF signaling in PC12 cells. Shoc2 overexpression renders longer-lasting ERK phosphorylation after EGF stimulation, with a resultant increase in neurite outgrowth. We also found evidence that knockdown of endogenous Shoc2 by specific shRNA leads to statistically significant inhibition of NGF-induced PC12 cell differentiation. Our data indicate that the scaffold protein Shoc2 contributes to NGF and EGF-induced neurite outgrowth in PC12 cells.

## Material and Methods

### Cell lines

HEK293T cells [18] were maintained in DMEM (Invitrogen, Carlsbad, CA) supplemented with 10% fetal calf serum (FCS, Invitrogen); these cells were analyzed after bFGF or EGF stimulation as described [18], [19]. PC12 rat pheochromocytoma cells [1] were cultured on collagen-coated plates using DMEM supplemented with 7.5% FCS, 7.5% horse serum (Invitrogen), 2 mM glutamine and 1% penicillin/streptomycin. PC12 cells were allowed to differentiate for 1-3 days by adding NGF (100 ng/ml) or EGF (100 ng/ml).

### Antibodies and reagents

Rabbit polyclonal antibodies to ERK (ERK1/ERK2) were purchased from Santa Cruz Biotechnology (Santa Cruz, CA); anti-phospho-ERK protein, anti-MEK (MEK1/MEK2), and anti-p-MEK1/2 were purchased from Cell Signaling Technology (Beverly, MA), and anti-tubulin was from Covance (Princeton, NJ). Monoclonal antibodies Anti-C-RAF, anti-pan-Ras, and anti-Grb2 were obtained from BD Transduction Laboratories (Franklin Lakes, NJ), whereas rabbit polyclonal anti-Shoc2 was produced and purified in the lab. Anti-HA and AU5 monoclonal antibodies (mAb) were purchased from Berkeley Antibody Company (Berkeley, CA). Anti β-actin mAb, and recombinant human basic fibroblast growth factor (bFGF), nerve growth factor (NGF), and epidermal growth factor (EGF) were from Sigma-Aldrich (St. Louis, MO). Anti-mouse and rabbit HRP-conjugated mAb were from GE Healthcare Bio-Sciences (Piscataway, NJ).

### DNA constructs

The plasmids pCEFL-KZ-HA, pCEFL-KZ-AU5, and pCEFL-KZ-HA-ERK (1 and 2), have been described [18], [19]; the pEGFP-C1 plasmid was purchased from Invitrogen. The plasmid containing hSur8 was kindly provided by Dr. Min Han (University of Colorado, Boulder, CO), and Shoc2 cDNA was amplified by PCR using specific primers providing Bam HI and Not I sites at the 5′ and 3′ ends, respectively. The amplified products were then subcloned into Bgl II and Not I sites within pCEFL-KZ-AU5. SureSilencing plasmids containing different shRNA for rat Shoc2 and GFP marker were purchased from SA Biosciences (NM_0011013155): plasmid n° 1 (aagctgtcaatcatgagtatt), plasmid n°2 (tgaaggacaatcagttaacat), plasmid n°3 (ggtgaactgtgtaacctcatt), and control plasmid (ggaatctcattcgatgcatac). Small interfering RNA (siRNA) for human Shoc2 were from Applied Biosystems (Carlsbad, CA). The oligonucleotides synthesized corresponded to the human Shoc2 encoding region of exon 8.

### Transient transfection assays

PC12 cells were nucleofected with shRNA plasmids using the Amaxa kit V (VCA-1003) following manufacturer's instructions. PC12 cells were transiently transfected with pCEFL-KZ-AU5-Shoc2 or pCEFL-KZ-HA-ERK-1 plasmids using Lipofectamine 2000 (Invitrogen) according to manufacturer's protocols. HEK293T cells were transiently transfected with pCEFL-KZ-AU5-Shoc2 or pCEFL-KZ-HA-ERK-1/ERK-2 plasmids using Jet-Pei (Polyplus-Transfection, Illkirch, France). All assays were performed 48 h post-transfection. The siRNA duplexes (100 nM) were transfected into HEK293T cells using X-treamGENE siRNA transfection reagent (Roche, Basel, Switzerland).

### Immunoprecipitation and immunoblotting

Immunoprecipitation and immunoblot analysis were performed as described [18], [19]. Protein extracts were resolved by SDS-PAGE, transferred to nitrocellulose membranes (Schleicher & Schuell) and probed with the appropriate antibody. Blots were developed by a peroxidase reaction using the ECL detection system (GE Healthcare Bio-Sciences).

### *In vitro* quantitative detection of intracellular phospho-ERK, total ERK and other kinases

Total ERK1/2, AKT, and GSK-3β and their phosphorylated forms phospho-ERK1/2 (pTpY185/187), phospho-AKT (pS473), and phospho-GSK-3β (pS9) were simultaneously quantitated by xMAP technology on a Luminex 200 platform (Bio-Plex-200, BioRad) using two independent panels custom developed and validated by Biosource. For these experiments, PC12 cells were lysed in Cell Extraction Buffer (Invitrogen) containing 10 mM Tris, pH 7.4, 2 mM Na_3_VO_4_, 1 mM EDTA, 100 mM NaCl, 1% Triton X-100, 1 mM EGTA, 20 mM Na_4_P_2_O_7_, 10% glycerol, 1 mM NaF, 0.5% deoxycholate, 0.1% SDS, with protease inhibitors (Complete from Roche and 1 mM PMSF). Cell extracts (2 µg) were analyzed in duplicate according to manufacturer's instructions. The concentration for each analyte was determined by interpolating the mean fluorescence intensity (MFI) for each sample from a standard curve using Bio-Plex System Software and a five-parameter logistic weighting algorithm.

### Neurite outgrowth assay

PC12 cells plated onto collagen-coated coverslips in 12-well dishes were transiently transfected and stimulated with NGF (100 ng/ml) or EGF (100 ng/ml) for 72 h. Cells were washed with phosphate-buffered saline before and after being fixed with 4% paraformaldehyde (10 min) and permeabilized with 0.2% Triton X-100 (10 min). Transfected cells were detected by GFP-fluorescence. Alternatively, cells were incubated with anti-AU5 mAb (1 h, room temperature). After several washes, cells were incubated with Alexa 488-conjugated secondary antibodies (Molecular Probes, Invitrogen; 1 h, room temperature). Cells (50 to 100/condition) were analyzed in a Nikon Eclipse TE200-U microscope. Cells with and without neurites were then counted. Total neurite length from each cell was measured using ImageJ software (National Institutes of Health). Image capture and analyses were performed blind to the experimental conditions. Neurite outgrowth was defined as a process equal to or greater than one cell body in length for EGF treatment, and equal to or greater than two cell bodies in length for NGF treatment.

### qRT–PCR

GoTaq qPCR Master Mix, from Promega, was used for real time qRT-PCR analyses. Gene specific pair primers were designed for *Shoc*2 (Forward: 5′-ATTGTGAGCTACATCCAGCG-3 and Reverse: 5′- TGCTTAGCATTCGAGAGAACA-3) and GADPH (Forward: 5′-AATGAAGGGGTCATTGATGC-3′ and Reverse: 5′-AAGGGAAGGTCGGAGTCAA-3′) with Primerdepot web. The Applied Biosystems 7500 fast apparatus was used to run the PCR performed as described previously [20]. In all cases, GADPH data were used for data normalization. Results were calculated by the 2-^ΔΔCT^ method.

### Statistical analysis

Data were analyzed statistically using the SPSS software (Chicago, IL). Results are displayed as mean ± SD of the corresponding number of experiments. Statistical significance was estimated by Student's *t* test for unpaired observations; P<0.05 was considered significant. For immunoblot analysis, we used linear correlations between increasing levels of protein and its signal intensity.

## Results

Ectopic overexpression of Shoc2 facilitates sustained ERK activation after stimulation by FGF or EGF

**Figure 1:**
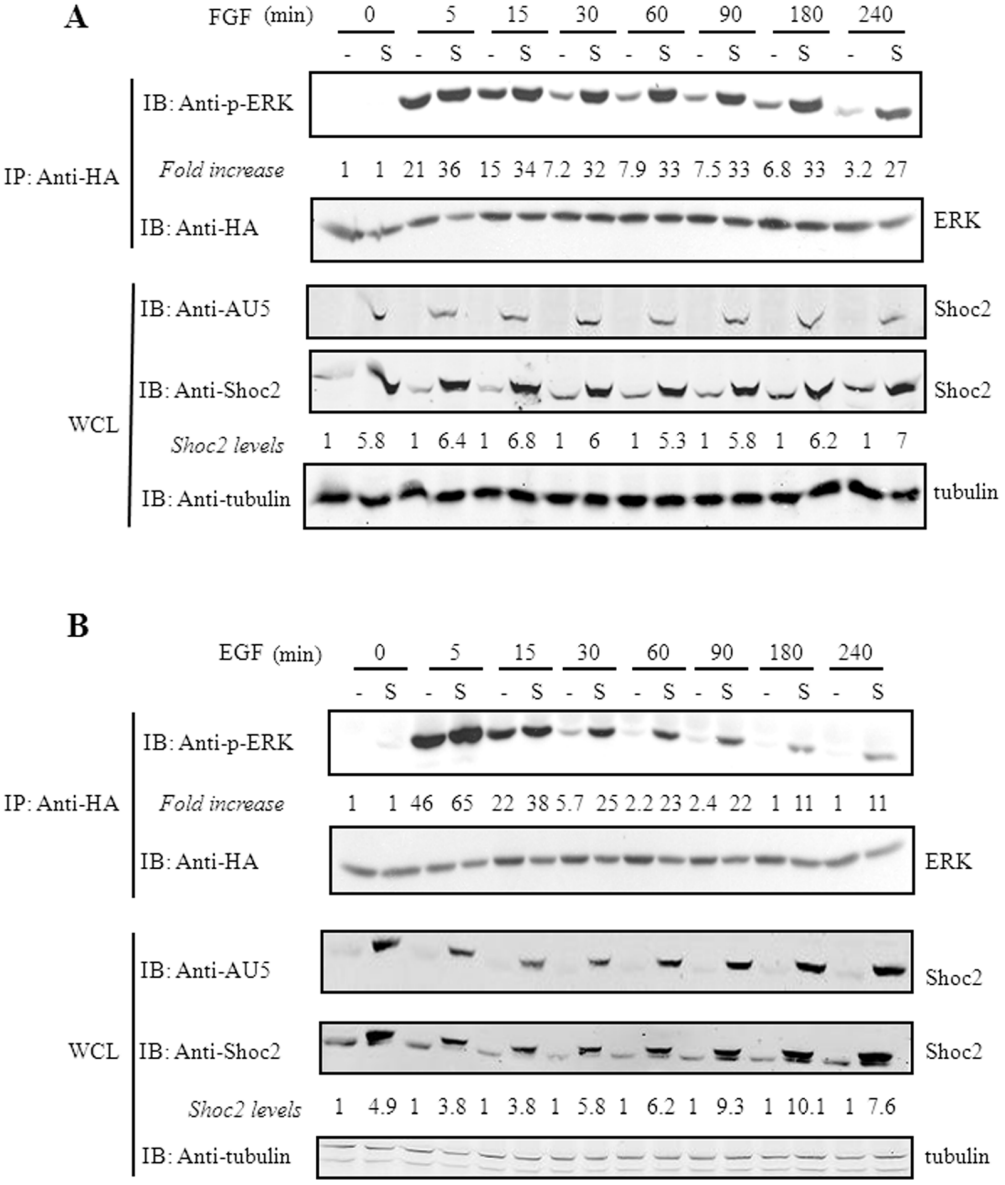
Shoc2 overexpression enhances the intensity and/or duration of RTK-elicited ERK activation. HEK293T cells transiently co-transfected with 1 µg pCEFL-KZ-HA-ERK1 and 2 µg pCEFL-KZ-AU5-hShoc2 (S) or 2 µg pCEFL-KZ-AU5 (-) were serum-starved for 18 h and then incubated with vehicle (0), 25 ng/ml FGF (**A**), or 10 ng/ml EGF (**B**), for 5 to 240 min. Cell lysates were immunoprecipitated with anti-HA antibody and analyzed by immunoblot using anti-p-ERK and -HA antibodies, to determine p-ERK1 and total HA-ERK1. Fold increase denotes the ratio p-ERK/ERK levels estimated as the mean of three separate assays (in all cases with a SD ≤10% of the mean). Expression levels of transfected AU5-hShoc2 constructs were detected by immunoblotting whole cell extracts (WCL) with the appropriate anti-AU5 antibody, and total Shoc2 expression was assessed by re-blotting of the same filters against anti-Shoc2 rabbit polyclonal antibody and using tubulin detection as control. In each point-time, the protein levels of Shoc2 were normalized versus tubulin amount and vector sample (-). Results were similar in three independent experiments.

Shoc2 has a synergistic effect on activation of the Ras-Raf-MEK-ERK pathway [12] and is required for rapid Ras interaction with Raf following EGF stimulation [13]. Here we examined whether Shoc2 overexpression affects the duration of ERK signaling induced by the stimulation of receptor tyrosine kinases (RTK). For this purpose, we stimulated HEK293T cells (transiently transfected with HA-ERK1 and AU5-hShoc2) with FGF or EGF, and measured ERK phosphorylation at different times. Shoc2 overexpression (with an approximate sixfold increase averaged over the endogenous Shoc2) raised not only the ERK activation levels triggered by FGF (Fig. 1A) or EGF (Fig. 1B), but also the duration of this activation. The kinetics of FGF-induced ERK activation was similar with or without Shoc2 overexpression but values increased when Shoc2 was overexpressed (Fig. 1A), whereas phospho-ERK levels were still detected 4 h post-EGF stimulation in Shoc2-transfected cells but for only 30 min in vector-expressing cells (Fig. 1B). These results suggest that Shoc2 proteins positively regulate the intensity and/or the duration of ERK activation elicited by FGF and EGF stimulation.

**Figure 2:**
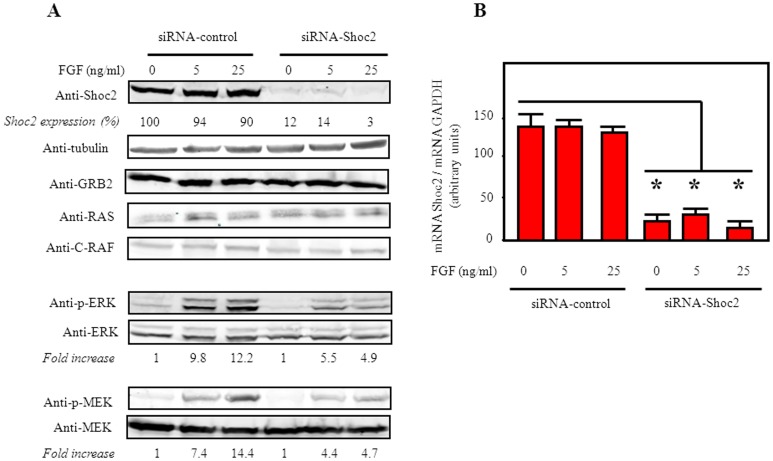
Shoc2 depletion reduces phospho-ERK and phospho-MEK levels induced by RTK activation. HEK293T cells were transiently transfected (100 nM) with either control siRNA (siRNA-Control) or human Shoc2-specific siRNA (siRNA-Shoc2). At 48 h post-transfection, cells were serum-starved for 18 h and then incubated with vehicle (0), or 5-25 ng/ml FGF for 10 min. (**A**), endogenous Shoc2 expression was assessed, from the cell lysates, by immunoblot using anti-Shoc2 rabbit polyclonal antibody and normalized to tubulin levels, expressed as a percentage (corresponding to the mean of five separate assays, in all cases with a SD ≤10% of the mean). The specific effect of the siRNA-Shoc2 was tested throughout detection of Grb2, Ras, C-Raf, ERK and MEK protein levels, by immunoblot of the cell lysates with the corresponding antibodies. Cell lysates were also analyzed by immunoblot using anti-p-ERK/ERK, and anti-p-MEK/MEK antibodies (bottom panels); in each case, fold increase denotes the ratio p-ERK/ERK, and p-MEK/MEK levels estimated as the mean of five separate assays (in all cases with a SD ≤10% of the mean). (**B**), the expression of Shoc2 was assessed by qRT-PCR. Data represents the mean ± SD of four independent experiments. The detection of GAPDH (normalization control) was performed using specific primers. Results were calculated by the 2-^ΔΔCT^ method. *P≤0.001 compared with siRNA-control; bars show SD.

To assess the relevance of Shoc2 in the RTK-ERK pathway, we transfected the above cells with siRNA oligonucleotides for Shoc2. A sharp reduction was observed both, in the expression of Shoc2 protein levels (Fig. 2A), and the amount of mRNA (Fig. 2B), compared to HEK293T cells transfected with control siRNA. This effect was clearly Shoc2-specific, since we show no alteration in the protein levels of other ERK activation-pathway members such as Grb2, Ras, Raf, MEK, and ERK (Fig. 2A). Shoc2 knockdown reduced phospho-ERK levels after stimulation with FGF (Fig. 2A), or EGF (data not shown), at a rate proportional to the decrease in Shoc2 levels. In addition, a similar reduction rates were observed in phospho-MEK levels (Fig. 2A). The effects of Shoc2 knockdown on FGF/EGF-induced MEK and ERK activation support the role of Shoc2 as an essential positive-regulator of the RTK-ERK pathway.

### Ectopic overexpression of Shoc2 enhances EGF-dependent PC12 cell differentiation

**Figure 3:**
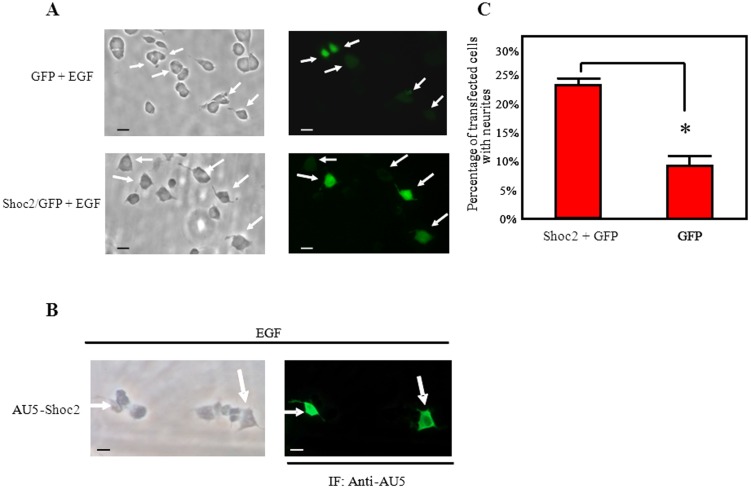
Shoc2 overexpression increases EGF-induced PC12 differentiation. **A**), PC12 cells were co-transfected with plasmids pCEFL-KZ-GFP (0.3 µg) and pCEFL-KZ-AU5 (0.7 µg) (top: GFP + EGF) or pCEFL-KZ-GFP (0.3 µg) and pCEFL-KZ-AU5-hShoc2 (0.7 µg) (bottom: Shoc2-GFP + EGF). Transfected PC12 cells were stimulated with EGF (100 ng/ml) for 72 h, fixed, and the presence of extending neurites was analyzed in GFP-positive cells by fluorescence microscopy (right: white arrows). Total cells were visualized with transmitted-light (left). A process equal in length or greater than one cell body was considered a neurite; bars, 10 µm. PC12 cells co-transfected with pCEFL-KZ-GFP and pCEFL-KZ-AU5-hShoc2 but not stimulated with EGF or NGF did not have neurites (not shown). **B**), PC12 transfected cells (as in A) were stimulated with EGF (100 ng/ml) for 72 h, fixed. Cells were incubated with anti-AU5 monoclonal antibody for 1h at room temperature and, after several washes, were incubated with Alexa 488-conjugated secondary antibodies. The presence of extending neurites was analyzed with transmitted-light (left-picture). PC12 cells overexpressing exogenous Shoc2 were detected as AU5-positive cells by fluorescence microscopy (right-picture and white arrows). A process equal to or greater than one cell body in length was considered a neurite; bars, 10 µm. **C**), histograms represent the percentage of transfected PC12 cells (GFP-positive: GFP vs Shoc2-GFP) with neurites after EGF treatment of panel A; values are the mean of three separate assays, in which at least 50 GFP-positive cells/assay were analyzed (*P<0.01); bars show SD.

ERK signaling duration is a limiting step for the different biological effects of EGF versus NGF in PC12 cells [2], [5], [6]. It is tempting to speculate that anything that increases the duration of EGF-dependent ERK activity could modify the EGF effects in PC12 cells. Since Shoc2 overexpression increased the duration of EGF-induced ERK activation (Fig. 1B), we tested whether Shoc2 overexpression in PC12 cells modified the EGF-induced biological fate of these cells. We compared the percentage of cells showing neurites after EGF treatment in PC12 cells transfected with GFP + AU5-hShoc2 versus GFP + empty vector (Fig. 3A-B). The percentage of GFP-positive PC12 cells showing neurite outgrowth after EGF treatment was significantly increased in cells overexpressing Shoc2 (Fig. 3C). Ectopic overexpression of Shoc2 alone (without EGF or NGF stimulation) was unable to elicit PC12 differentiation (not shown). These results suggest that Shoc2 protein modulates the outcome of EGF signaling on PC12 differentiation.

### Shoc2 downregulation reduces both NGF-induced PC12 differentiation and ERK activation

**Figure 4:**
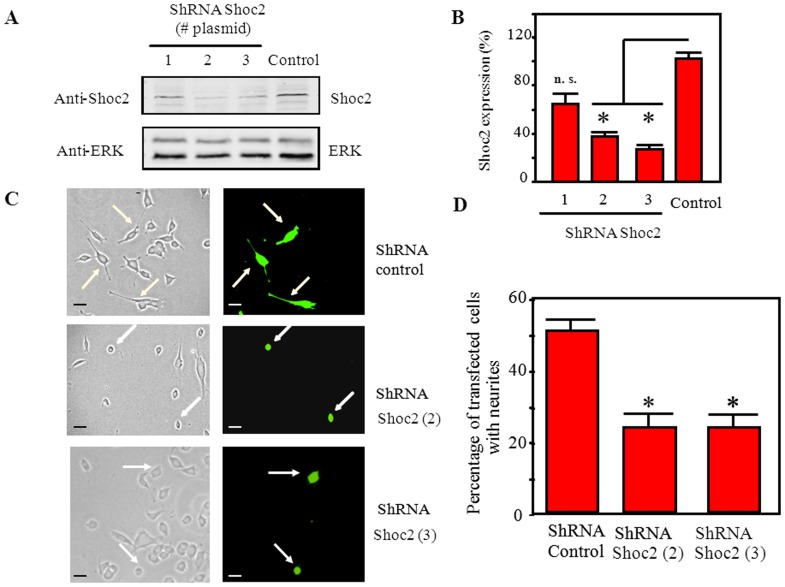
Shoc2 knockdown reduces NGF-elicited of PC12 cell differentiation. A), PC12 cells were nucleofected with shRNA-control-GFP plasmid (ShRNA Control) or specific rat shRNA-Shoc2-GFP plasmids (ShRNA Shoc2 #1-3). At 48 h post-transfection, cell lysates were analyzed by immunoblot. B), Shoc2 levels determined using anti-Shoc2 polyclonal antibody were normalized to total ERK levels, and expressed as a percentage (right). Results were similar in two additional experiments. *P≤0.001 and n.s. (not significant) compared with siRNA-control; bars show SD. C), PC12 cells nucleofected with either shRNA-control-GFP plasmid (ShRNA Control) or specific rat shRNA-Shoc2-GFP plasmids (ShRNA Shoc2 (2) and (3)) were stimulated with 100 ng/ml NGF for 72 h. After fixing, GFP-positive cells were detected by fluorescence microscopy (right: arrows) and total cells were visualized with transmitted light (left). Extending processes equal in length or greater than two cell bodies were considered neurites; bars, 10 µm. D), histograms represent the percentage of transfected PC12 cells (GFP-positive) with neurites after NGF treatment; values are the mean of three separate assays, in which at least 50 GFP-positive cells/assay were analyzed (*ShRNA Shoc2 (2 or 3) vs ShRNA Control; P<0.01), bars show SD.

**Figure 5:**
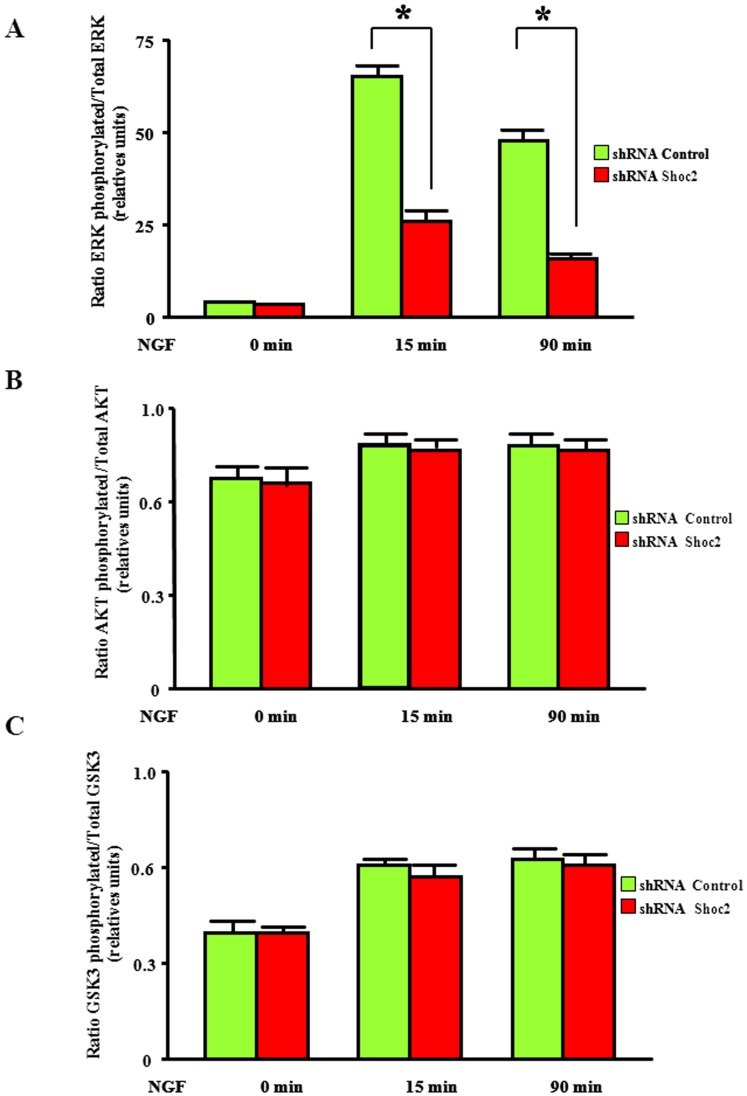
Shoc2 knockdown reduces NGF-induced ERK activation in PC12 cells. PC12 cells nucleofected with shRNA-control-GFP plasmid (ShRNA Control) or specific rat shRNA-Shoc2-GFP #3 plasmid (ShRNA Shoc2) were stimulated with 100 ng/ml NGF for the times indicated. In each assay, 2 µg PC12 cell extracts were analyzed by xMAP technology in a Luminex-200 platform for quantitative and simultaneous detection of phospho-ERK (pTpY183/185) and total ERK protein (Panel **A**), phospho-AKT and total AKT (Panel **B**) or phospho-GSK3 and total GSK3 protein (Panel **C**). Histograms show the ratio of p-ERK/ERK (Panel **A**), p-AKT/AKT (Panel **B**) or p-GSK3/GSK3 levels (Panel **C**) after the indicated time of NGF stimulation; values are the mean of three separate assays performed in duplicate (*P≤0.001), bars show SD.

Shoc2 overexpression in PC12 cells had no effect on NGF-stimulated neurite outgrowth (not shown), probably due to the highly sustained NGF-dependent ERK activation in these cells [2], [5], [6]. Shoc2 could nevertheless be an important factor in NGF-induced PC12 differentiation, given its role in RTK-dependent ERK activation (Figs. 1 and 2). To test Shoc2 effects on PC12 cell differentiation, we downregulated their endogenous levels by shRNA. We transfected PC12 cells with various bi-cistronic expression plasmids carrying specific shRNA to silence rat Shoc2, together with cDNA for GFP, and performed a morphological analysis of transfected GFP-positive/shRNA PC12 cells. We observed a sharp reduction in Shoc2 protein levels in cells transfected with plasmids 2 and 3 (70% and 80%, respectively) as compared to cells transfected with control shRNA (Fig. 4A–B). Analysis of NGF-induced neurite outgrowth in PC12 cells transfected with shRNA-Shoc2 expression plasmids (2 and 3) or with control shRNA (Fig. 4C) showed that shRNA-Shoc2 significantly decreased the percentage of cells with neurites (Fig. 4D). These results indicate that Shoc2 knockdown blocks NGF-promoted PC12 cell differentiation. This reduction of Shoc2 protein levels in PC12 cells also correlated with lower ERK activation after NGF stimulation (Fig. 5A), but had no effect on other kinases such as AKT or GSK3 (Fig. 5B–C), supporting Shoc2 specificity in the ERK pathway. These results show that Shoc2 is necessary for appropriate ERK activation and neural differentiation of PC12 cells triggered by neurotrophins such as NGF.

## Discussion

The extracellular signal-regulated kinase (ERK) signaling pathway is functionally relevant in converting extracellular stimuli into a wide range of cell responses, including proliferation, differentiation, and survival of normal and malignant cells [2], [21], [22], [23]. ERK-(1/2) are activated by mitogens and are usually upregulated in human tumors [24] and in a class of developmental disorders, referred as RASopathies, such as Noonan and LEOPARD syndromes [25]. In this pathway, Ras (H-, N- or K-) activation by GTP-loading elicits the plasma membrane recruitment of Raf (A-, B- or C-) that phosphorylates and activates to the dual specificity kinases MEK1/2, which then subsequently phosphorylates and activates ERK1/2. The activity at each level of the pathway is fine-tuned through different control steps, which then provoke the specificity of biological outcomes, both throughout assembling signaling complexes at particular subcellular regions, and by the control of signal dynamics [26], [27]. Among these ERK-pathway regulators are the scaffold proteins, that tether and target signaling-complexes to different subcellular locations [27], [28], [29]. These scaffolds include the kinase suppressor of Ras1 (KSR1), paxillin, MEK partner 1 (MP1), IQ motif containing GTPase activating protein 1 (IQGAP1), caveolin-1 [30], [31], and more recently to Shoc2.

The scaffold protein Shoc2 is conserved in all metazoans, with high binding affinity to Ras and Raf proteins and a synergistic effect on RTK activation of the ERK signaling pathway [12]. Shoc2 was firstly found in *C. elegans* as a positive regulator of the Ras-pathway [10], [11], and this protein contains almost exclusively leucine-rich repeats giving a curved solenoid structure suited for protein-protein interaction [32]. The Shoc2 N-terminal domain binds to M-Ras and C-Raf, whereas the C-terminal region of Shoc2 is responsible for Shoc2 targeting to late endosomes [33]. Independently of the mechanism of action considered, either accelerating Ras-Raf binding [12], [13], [14], or by Raf S259 dephosphorylation throughout a ternary complex M-Ras/Shoc2/PP1c [16], [17], Shoc2 is essential for the activation of Raf. In this context, Shoc2 expression is able to modulate both ERK activation and resistance to apoptosis of B-Raf^V600E^/N-Ras^Q61K^ melanoma cells in the presence Raf inhibitor [34]. In addition, a naturally-occurring human Shoc2 mutant was described that yields high phospho-ERK levels, causing a Noonan-like syndrome [15]; this gain-of-function mutation in Shoc2 (S2G) generates an aberrant N-myristoylation site, resulting in a Shoc2 protein constitutively plasma membrane targeted [15] and unable to translocate to late endosomes [35].

Here, we demonstrate that Shoc2 is an essential protein for ERK activation, dependent on RTK stimulation. Our results show that downregulation of endogenous Shoc2 expression (through specific siRNA and shRNA) significantly reduced phospho-ERK levels after stimulation by FGF, EGF or NGF. Although the role of Shoc2 on ERK activation in the EGFR signaling pathway has been described [13], [16], [36], we found the same behavior for Shoc2 in other cell systems and for other RTK-dependent signaling routes, such as fibroblast growth factor-receptor (FGFR) and tropomyosin-related kinase (Trk) families. Our results not only are concur with the description of a Shoc2 mutant involved in a Noonan-like syndrome by deregulated ERK activation [15], but also with inhibition of the Ras-ERK pathway by Erbin, another protein rich in leucine domains that blocks Shoc2 interaction with Raf and Ras, thus down-regulating ERK activation through molecular binding between Erbin and Shoc2 [37], [38] that is enhanced by Desmoglein-1 [39].

Based on to the critical role of Shoc2 in the RTK-ERK pathway, we showed that ectopic overexpression of Shoc2 in epithelial cells was able to increase phospho-ERK levels evoked by FGF or EGF. We also found that Shoc2 overexpression produced persistent ERK activation, with an increase in duration of the effect of ≥240 min post-EGFR stimulation. Evidences indicate that differences in ERK activity duration produce variations in signaling that regulate cell fate. ERK signaling duration is a limiting step for the different biological effects of EGF versus NGF in PC12 cells [1], [2]. In this cell system, EGF induces rapid, transient ERK activation, eliciting proliferation rather than neurite outgrowth [2]; in contrast, NGF induces rapid, sustained ERK phosphorylation that leads to PC12 differentiation [2], [5], [6]. It is tempting to speculate that anything that increases the duration of EGF-dependent ERK activity could modify the EGF effects in PC12 cells. Shoc2 overexpression produces more persistent EGF-dependent ERK activation, and also enhances EGF-induced neurite outgrowth in PC12 cells. Although this biological effect is not dramatic, the results obtained were statistically significant and are in agreement with the idea that duration of ERK activity is critical for PC12 cell fate.

Our data suggest that endogenous Shoc2 could also be implicated in differentiation processes. Although Shoc2 overexpression in PC12 cells had no effect on NGF-stimulated neurite outgrowth, probably due to the highly sustained NGF-dependent ERK activation in these cells [2], [5], [6], Shoc2 knockdown significantly reduced both NGF-induced ERK activation and neurite outgrowth. Our data therefore indicate that the Shoc2 scaffold protein is essential for appropriate ERK activation and neural differentiation of PC12 cells triggered by neurotrophins such as NGF. These results could be considered opposite to other studies, where Shoc2 has been proposed as an anti-differentiation factor that stimulates proliferation for maintenance of self-renewal in neural progenitors cells (NPC) through modulation of the Ras-ERK pathway [40]. However, it is known that the Ras-ERK pathway elicits both inhibition of differentiation and activation of proliferation in NPC [40], [41]. Obviously, PC12 are not biologically comparable to NPC, but in both cases Shoc2 is absolutely critical for the sustained functionality of the Ras/Raf/MEK/ERK pathway. Shoc2 probably is necessary to maintain the proliferation and preventing differentiation in stem cells (especially in response to bFGF and EGF) [39], [40]; but either in other lineage cells (such as PC12, with higher differentiation level), or with other stimuli (such as NGF), the Shoc2 role can be different. Same situation occurs with Spry2 (other docking/scaffold protein modulator of the ERK pathway): while Spry2 inhibits the differentiation of PC12 pheochromocytoma cells in response to FGF [42], it promotes FGF-induced differentiation of C2C12 myoblasts [43], or it is involved in the emergence of biased EGF signaling in the progeny of dividing neural stem cells [44]. All these data (including our results) highlight the complex network of molecular interactions stablished among the Ras/Raf/MEK/ERK pathway and its docking/scaffold protein modulators, according to each cell lineage context and the response to different stimuli.

The observation of the same number of GFP-PC12 positive cells at the end of the differentiation assay, independent of their transfection status with shRNA-control-GFP or shRNA-Shoc2-GFP plasmids (not shown), suggests that Shoc2 downregulation does not affect PC12 cell viability. These results concur with previous reports indicating that PC12 cell survival does not require Ras-ERK pathway activity, but it is instead dependent on PI3K signaling [45] which is unaltered by Shoc2 knockdown (Fig. 5).

The role of Shoc2 in differentiation processes could explain both the effects of a dominant mutant form of this protein in developmental disorders such as the Noonan-like syndrome [15], and the defects of the atrioventricular canal that cause early embryonic lethality when the *Shoc2/Sur8* gene is inactivated in mouse endothelial cells [46]. Besides being a scaffold protein essential for the RTK-dependent ERK activation pathway, Shoc2 could have other roles due to its presence in nuclei [15] that we have observed during PC12 differentiation (Martinez N et al., manuscript in preparation).

In conclusion, our data support positive modulation by Shoc2 of RTK-induced ERK activation. The functional role of this protein could be relevant in the context of variations in Ras-Raf-ERK signaling that regulate cell differentiation processes. Future studies might show Shoc2 protein to be useful as target for the design of new therapeutic drugs for several diseases.
